# Navigating chest pain online: who uses health information and what actions follow?

**DOI:** 10.1186/s12875-025-03110-7

**Published:** 2025-11-25

**Authors:** Eva D. van Dijkman, Ysette K. de Boer, Ellen L. de Hollander, Mattijs E. Numans, Hans-Marc J. Siebelink, Tobias N. Bonten

**Affiliations:** 1 Department of Public Health and Primary Care, Leiden University Medical Center, Postzone V0-P, Postbus 9600 , Leiden, 2300 RC The Netherlands; 2 Department of Research and Development, Cluster Thuisarts, Dutch College of General Practitioners, Utrecht, 3528 BL The Netherlands; 3 Department of Cardiology B4-17, Leiden University Medical Center, Postbus 9600, Leiden, 2300 RC The Netherlands

**Keywords:** Chest Pain, Internet, Health Behaviour

## Abstract

**Background:**

Online health information websites, like Thuisarts.nl, guide patients with chest pain toward appropriate care and support sustainable healthcare use.

**Objectives:**

This study explores the demographics, symptoms, actions, and decision-making of individuals using Thuisarts.nl for chest pain.

**Methods:**

A cross-sectional questionnaire was carried out (February 2022–October 2023) via pop-ups on two Thuisarts.nl pages about chest pain. Adults seeking chest pain information for themselves were invited to complete an anonymous questionnaire. Responses were categorized based on whether participants sought immediate care or waited to contact a healthcare provider. Descriptive statistics summarized participant characteristics, and logistic regression examined the association between cardiac-like symptoms and immediate care-seeking, adjusted for age, sex, and comorbidity.

**Results:**

Of the 791 participants who started the questionnaire (response rate 10%), 66% were female, with a mean age of 52 years. Most participants (78%) reported non-anginal pain, while 5% described symptoms resembling typical angina. Participants with cardiac-like symptoms were more likely to seek immediate care than those with non-cardiac pain (OR 4.67, 95% CI 1.33–16.42, *p* = 0.016). Overall, 83% of participants found the information helpful; of these, 14% sought immediate care, while 86% waited before contacting a healthcare provider.

**Conclusion:**

Thuisarts.nl for chest pain is primarily used by a low-risk group and is helpful to most visitors. While many wait to contact a healthcare provider after reading the website, those with cardiac-like symptoms tend to seek immediate care. These findings highlight the importance of providing clear, accessible information in supporting sustainable chest pain management.

**Supplementary Information:**

The online version contains supplementary material available at 10.1186/s12875-025-03110-7.

## Background

Online websites are increasingly used by patients seeking information about their health issues and deciding whether to consult a doctor. In 2012, the Dutch College of General Practitioners (NHG) launched Thuisarts.nl, an evidence-based website providing reliable and understandable medical information. Thuisarts.nl is public, free for everyone and used at home as well as during GP consultations [1]. Similarly, the NHS website in the UK serves a comparable purpose [1, 2]. Both aim to support informed decision-making and guide patients on when to seek medical assistance.

The content of the website is based on 45 medical guidelines and additional consensus-based guidelines regarding self-care, offering visitors information on various health topics [3]. Thuisarts.nl is one of the most visited healthcare websites in the Netherlands (18 million inhabitants), with 75 million page views annually. The ‘Chest pain’ pages are among the most popular, with 533.000 unique page views per year, reflecting a widespread concern about this symptom [4, 5].

Chest pain can have different causes, varying from relative benign conditions (e.g. musculoskeletal origin or gastro-oesophageal reflux disease) to potential life-threatening events such as coronary artery disease (CAD; i.e. angina pectoris or myocardial infarction), aortic dissection and pulmonary embolism [6–8]. While CAD is a key concern, its prevalence in primary care is relatively low (1.5–10%) [6, 9].

In multiple Western healthcare systems, general practitioners (GPs) serve as gatekeepers to secondary care. Chest pain accounts for 0.7% to 2.7% of all GP visits [6, 7, 10]. In the Netherlands, this equals 558.000 to 2 million chest pain consultations each year [11]. Emergency department (ED) data further emphasize its burden, with 2.25 million ED visits recorded in 2019 in the Netherlands, 14% of which were for chest pain [12]. These numbers highlight the impact of chest pain on healthcare services, a challenge expected to grow with an aging population [13].

Previous studies suggest that reliable online health information can reduce primary care consultations [3, 14]. For example, Thuisarts.nl has been shown to decrease GP visits for common health issues (e.g., constipation, low back pain, sinusitis), but chest pain was not specifically included [3]. So, Thuisarts.nl has the potential to help individuals with chest pain to make better informed decisions and possibly reduce pressure on the health care system, GP consultations and ED visits. However, there is currently no data about how individuals use Thuisarts.nl for chest pain. Gaining insight into this is essential for improving chest pain information on healthcare websites and ensuring that healthcare services remain accessible. This study aims to explore which individuals use Thuisarts.nl for chest pain, how they interpret the information, and what actions they take based on it.

## Methods

### Study design and setting

We carried out a cross-sectional anonymous questionnaire on Thuisarts.nl from February 2022 to October 2023 (see Supplementary File). The website provides information on prevention, self-care, diseases, treatments, and examinations for both primary and secondary care. The questionnaire was available on two pages: “I have chest pain” and “I have angina”, covering causes of chest pain, symptoms, and when to seek immediate care. A pop-up button linking to the questionnaire was placed on both pages, and it was completed in the secure Castor Electronic Data Capture (EDC) environment. Participants had the option to leave the questionnaire at any point. The questionnaire was written in Dutch at language level B1, matching the website content, and was assessed by a linguist.

All visitors to the subpages were able to click on the link. Participants were included if they were 18 years or older and sought information about chest pain for themselves, as determined by the first questions. No distinction was made between acute and non-acute chest pain, as patients generally don’t differentiate between those medical entities. The goal was to include 450 visitors.

### Questionnaire and theoretical framework

The questionnaire was developed for this study and assessed participant characteristics (age, sex, history of cardiometabolic disease, chest pain classification, and characteristics of pain) without identifying information. Chest pain classification included constricting discomfort, provoked by physical exertion, and pain relieved by rest. The pain characteristics included the level of pain, its fast or slow onset, and additional complaints. It also explored whether the Thuisarts.nl information helped participants decide, what sections were most helpful, and what actions were taken. Participants were categorized into two groups: those who sought immediate care and those who waited to contact a healthcare provider. The group who waited includes those who contacted a healthcare provider after a few days and those who never contacted one, after reading the information on the website.

The Health Belief Model (HBM) was used to design part of the questionnaire to understand what behavioural determinants influence the decision to contact a healthcare provider. The HBM is a practical, theoretical framework and assumes that existing beliefs can predict future behaviours [15]. We included perceived severity, perceived susceptibility and worry. The determinant “worry” was added because previous studies on acute chest pain have shown that patients’ worry is an important predictor of experiencing a cardiac event [9, 10]. These constructs were measured with questions asking why participants either contacted a healthcare provider immediately or waited, with response options reflecting whether the information made them take their complaints more or less seriously (perceived severity), helped them understand potential health risks (perceived susceptibility), increased or decreased their concern (worry), or confirmed their suspicions (Supplementary File).

### Statistical analysis

IBM SPSS Statistics (version 29.0.0.0) for Windows was used to conduct all statistical analysis. Frequencies and percentages described dichotomous variables, and means with standard deviations described continuous variables. Logistic regression was used to calculate the odds ratio with a corresponding 95% confidence interval (CI), examining the association between pain resembling cardiac pain and the likelihood of seeking immediate contact with a healthcare provider, adjusted for age, sex, and the presence of any comorbidity (hypertension, diabetes mellitus, angina pectoris, claudication intermittens, myocardial infarction, TIA/CVA). Interactions between age, sex, and comorbidity were also tested. Data on other potential confounders, such as literacy level or worry scores from the HBM, were not available.

## Results

**Fig. 1:**
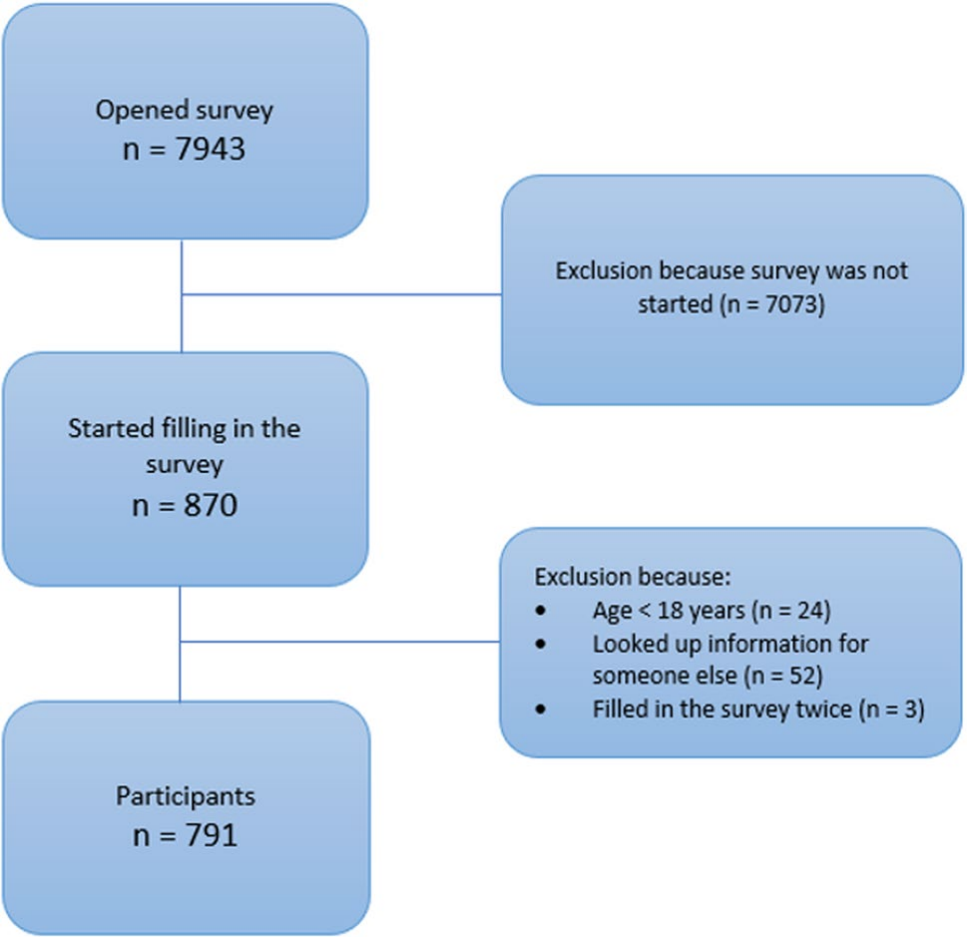
Inclusion of participants

Between February 2022 and October 2023 the survey was opened 7943 times, and 791 (10%) participants started the questionnaire (Fig. 1). Due to the optional nature of the questionnaire, the number of participants varied for each characteristic (Table 1). The mean age of the study population was 52 years (SD 21), ranging from 18 to 90 years of which the majority (66%) was female. A history of cardiometabolic conditions was reported in 28% of participants. The chest pain resembled non-anginal pain in 78% of the participants and only 5% had symptoms resembling typical angina pectoris. Analysis of other pain characteristics revealed that 52% were somewhat restricted in their activities due to the pain. Additional symptoms such as sweating, nausea, and dyspnoea were noted by 45% of participants (Table 1).

**Table 1:** Participant characteristics

	*n* (%)
Age (years) (*n*=791)*	
18–34	215 (27)
35–49	120 (15)
50–64	203 (26)
65+	253 (32)
Sex (*n*=381)*	
Male	128 (33)
Female	251 (66)
Other	2 (1)
History of cardiometabolic disease** (*n*=390)*	
None	280 (72)
Hypertension	53 (14)
Diabetes mellitus	27 (7)
Angina pectoris	32 (8)
Claudicatio intermittens	10 (3)
Myocardial infarction	9 (2)
TIA/CVA	16 (4)
Other	39 (10)
Classification of chest pain*** (*n*=459)*	
Typical angina (all 3 characteristics)	22 (5)
Atypical angina (2 of 3 characteristics)	78 (17)
Non-anginal pain (0 or 1 of 3 characteristics)	359 (78)
Characteristics of pain	
Level of pain (*n*=459)*	
-I could do everything I normally do	94 (21)
-I could do less than normal	240 (52)
-The pain was continuous or so bad I could not do a thing	125 (27)
Start of the pain (*n*=455)*	
-Slow	214 (47)
-Fast	241 (53)
Additional to chest pain** (*n*=471)*	
-Sweating	70 (15)
-Nausea	71 (15)
-Dyspnoea	82 (17)
-Fever	25 (5)
-Other	89 (19)
-None	260 (55)

* number of participants who filled in the question

** multiple answer options possible

*** characteristics: 1) constricting discomfort, 2) provoked by physical exertion, 3) relieved by rest

**Fig. 2:**
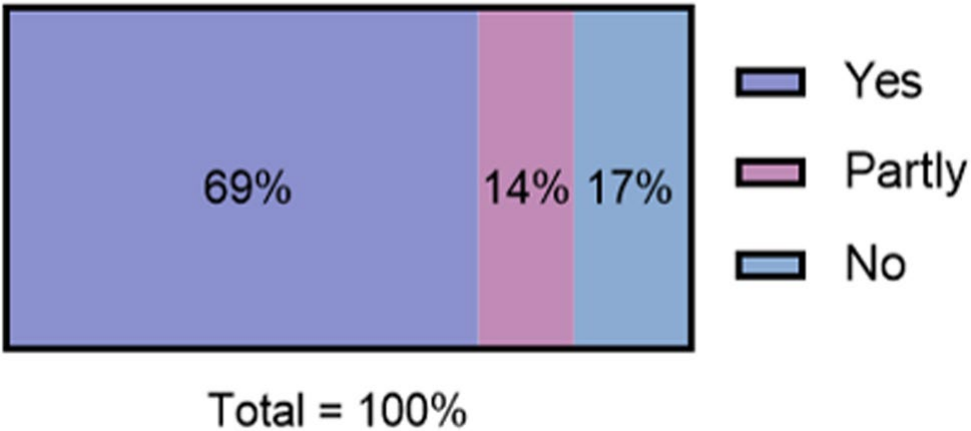
Did the information on the website help? (*n *= 669)

A total of 83% of participants found the information on Thuisarts.nl useful, with 69% rating it as useful and 14% as partly useful (Fig. 2). In contrast, 17% reported that the information had not helped at all (Fig. 2). Of this group, 90 participants provided additional feedback on why the website was not helpful. Among these, 43 (48%) indicated that their chest pain did not match the descriptions provided on the website, and 11 (12%) stated that they did not understand the information.

**Fig. 3:**
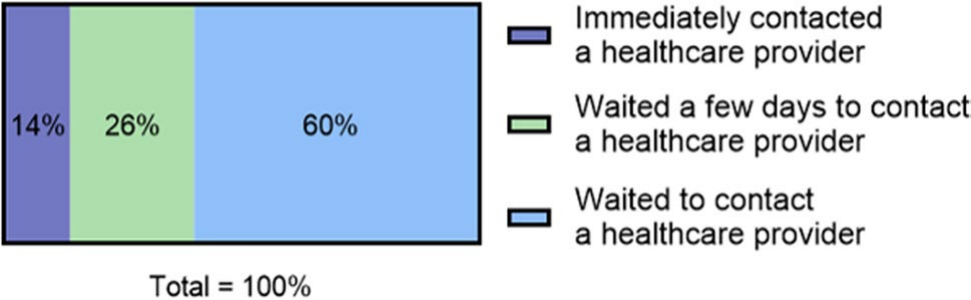
Action taken by participants after reading the information on the website (*n = *459)

Of those who reported that the information had helped, 14% contacted a healthcare provider immediately, 26% contacted a healthcare provider in the following days, and 60% chose not to contact a healthcare provider (Fig. 3). The logistic regression showed that individuals with symptoms resembling cardiac pain were significantly more likely to seek immediate contact with a healthcare provider. This association remained significant after adjusting for age, sex, and the presence of any comorbidity (OR 4.67, 95% CI 1.33–16.42, *p* = 0.016). No significant interactions were observed between age, sex, and comorbidity.

**Fig. 4:**
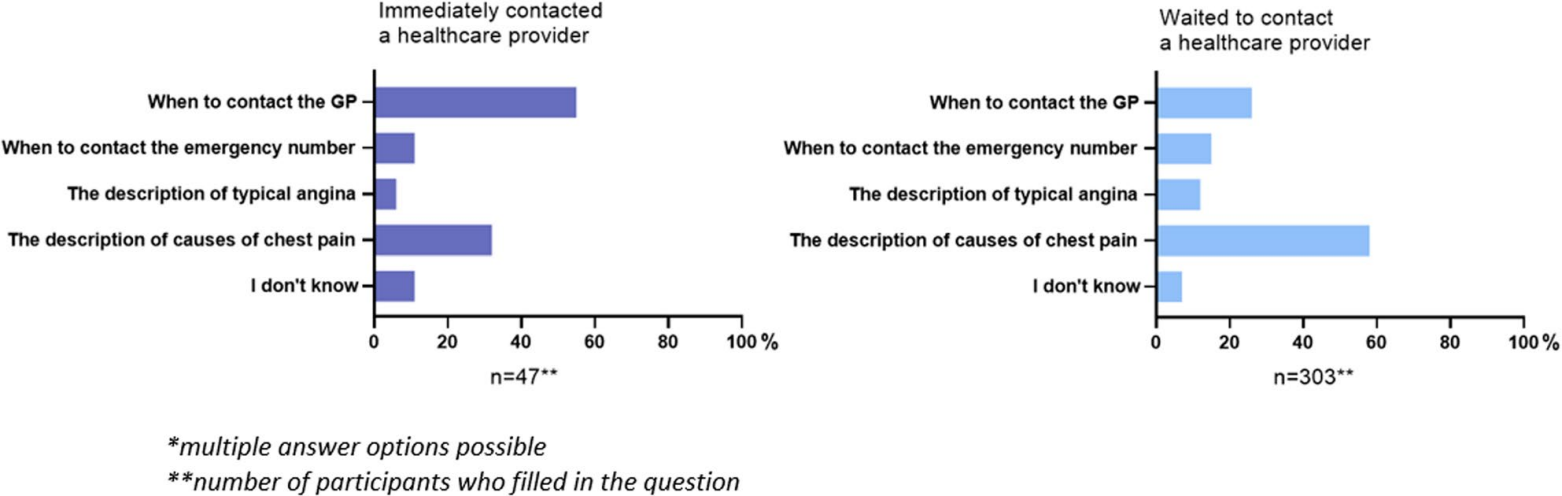
Most helpful sections of information on the website*

Two groups were compared: those who sought immediate contact and those who waited to contact a healthcare provider (waited a few days or did not contact one at all). Of those who contacted a healthcare provider immediately, 55% felt that the information under “Call your GP or the GP emergency service directly in these situations” was most helpful in guiding their decision to take action (Fig. 4). In contrast, 58% of those who waited to contact a healthcare provider, reported that the section titled “The description of causes of chest pain” contributed most to determine their next steps.

**Fig. 5:**
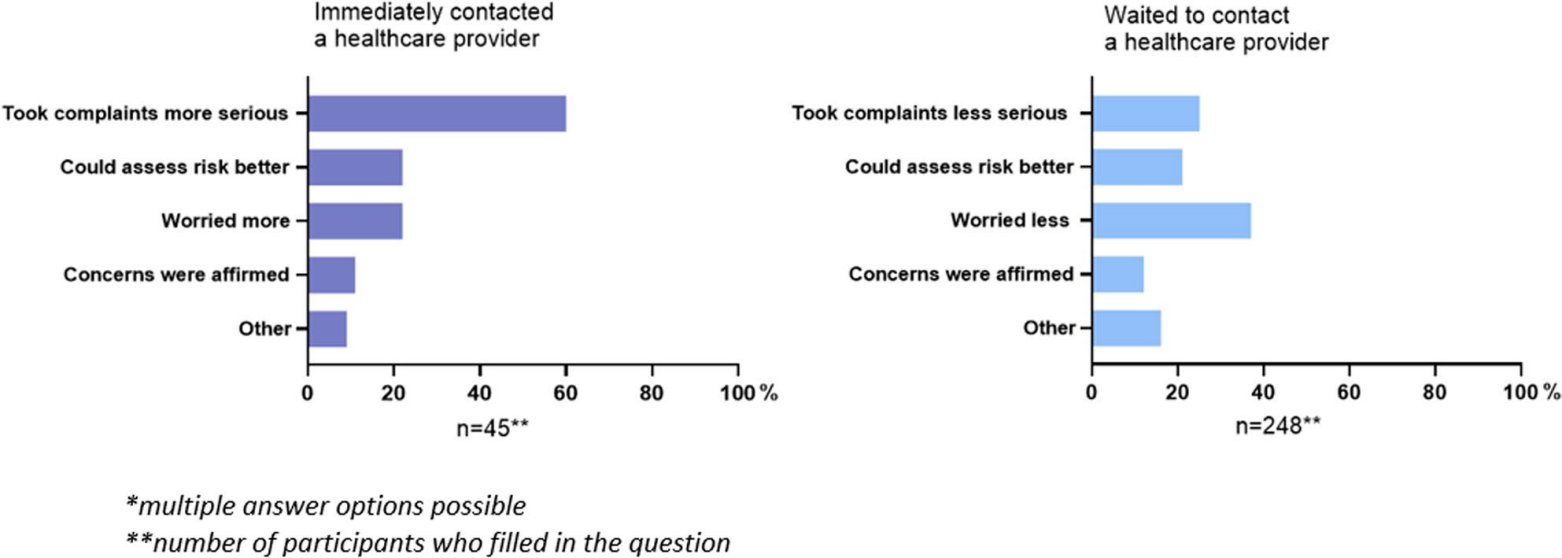
Why people took action after reading the information on the website*

The reasons for taking a certain action differed between the two groups (Fig. 5). Those who contacted a healthcare provider immediately took their symptoms more serious after reading the information on the website (60%). In contrast, among those who waited to contact a healthcare provider, the responses showed more variation: some individuals were less worried (37%) after reading the information, while others took their symptoms less serious (25%) or could assess the risk better (21%).

## Discussion

### Main findings

Our findings reveal that the participants seeking information on Thuisarts.nl for chest pain had few comorbidities and most reported non-anginal complaints, indicating that the group actively seeking online information is a population at low risk for acute coronary syndrome. Most participants found the information on Thuisarts.nl useful, and the majority decided to wait and monitor their symptoms before contacting a healthcare provider.

Our analysis also showed that individuals identifying their pain as cardiac related were more likely to seek immediate care, while those with non-anginal complaints were more likely to wait before contacting a healthcare provider. In particular, the sections titled “Call your GP or the GP emergency service directly in these situations” and “The description of causes of chest pain” were perceived as most helpful in making a decision. These findings support the aim of Thuisarts.nl to help individuals make well-informed decisions and take appropriate action when experiencing chest pain.

### Strengths and limitations

To our knowledge, this is the first study to examine the patients’ perspective on using a website before consulting a doctor for chest pain, providing valuable insights into how individuals approach online health information. However, several limitations must be considered. Selection bias is a concern, as the survey’s placement on a website may have selected individuals with higher literacy and internet access [16, 17]. Some participants reported difficulty understanding the B1-level Dutch text, which may limit the generalizability, particularly to the 12% of the Dutch population with low literacy [18].

Only approximately 10% of those exposed to the survey pop-up initiated the questionnaire, and fewer completed it, introducing potential self-selection and non-response bias [19]. This likely led to an underrepresentation of high-risk individuals as people with acute complaints may have been less likely to participate due to time constraints. On the other hand, it is reasonable to assume that in acute situations, people may not have the time or don’t want to search the internet, suggesting that those with non-acute complaints are more likely to visit the website, which aligns with our findings.

Additionally, participants could leave the questionnaire at any point, resulting in incomplete responses. Finally, the questionnaire asked about symptoms and subsequent actions, but these actions were not verified, potentially introducing recall bias or gaps between intended and actual behaviour. However, given the descriptive nature of the study, the responses obtained are considered adequate for our analysis.

This study focused specifically on the Dutch website Thuisarts.nl, which may limit the direct applicability of the findings to other countries. However, we expect that our results are generalisable to other Western countries, as patients in many settings seek online health information and use it to guide their decisions about whether to consult a healthcare professional.

Despite these limitations, the study offers valuable insights into how individuals with chest pain interact with online health resources and how these resources may influence decision-making in this context.

### Comparison with existing literature

Most literature on online health information tools focuses on decision-support tools that assist patients in determining whether to seek care, compared to traditional telephone triage. For example, a Dutch study on the app “Should I See a Doctor?” found it could guide patients in contacting out-of-hours primary care clinics for acute care. This app serves as an interactive decision tool (triage) [20]. Thuisarts.nl is not a triage tool, but an evidence-based health information platform covering prevention, self-care, and treatment across healthcare stages. It helps users make informed choices without providing specific triage advice.

Evidence suggests that the impact of online health tools is mixed: some studies indicate they reduce consultations by supporting self-management [3, 14, 21, 22], while others report an increase in GP consultations due to users’ concerns [23–25]. The key difference lies in the type of tool. Symptom checkers or systems with direct healthcare communication may increase consultations due to cautious triage advice. In contrast, evidence-based health information websites, aimed at a broad population, tend to reduce consultations by providing users with the information needed to manage symptoms independently. However, these previous studies did not specifically address chest pain.

These findings, showing that evidence-based health information websites reduce consultations, are directly applicable to our study as they demonstrate how platforms like Thuisarts.nl influence patients’ decisions regarding chest pain. Specifically, our study suggests that users of Thuisarts.nl often felt reassured by the information provided, allowing them to manage their chest pain symptoms effectively without immediate consultation. Most participants with non-anginal complaints opted to wait and monitor their symptoms, while those with cardiac-related pain were more likely to seek immediate care. These findings align with and support existing evidence indicating that reliable health information can reduce healthcare consultations and lead to appropriate referrals in cases of more serious conditions [3, 14].

### Clinical implications and future directions

This study highlights the value of evidence based, open-access health care information websites for individuals with chest pain. The information on Thuisarts.nl ensures that people can make better-informed decisions about their care. In this way, the chest pain pages of the Thuisarts.nl website probably contribute to the right care at right place and at the right time, optimizing the use of healthcare resources. This is a crucial point in the patient’s healthcare journey, as it plays a vital role in helping people navigate their symptoms and make informed decisions about seeking care.

However, there are areas for improvement in Thuisarts.nl for chest pain: we recommend positioning the most useful sections near the top of the page. The section on “When to contact your GP immediately” is already appropriately placed, whereas the “The pain resembles the description of…” section could be moved up, as it was particularly helpful for the largest group of participants, who chose to monitor their symptoms. Additionally, it is important to consider individuals who struggle with reading or comprehension. Incorporating illustrations and videos could effectively reach those with low literacy and language barriers [26, 27].

This study explored the population with chest pain complaints using Thuisarts.nl, their interpretation of its information and subsequent actions. Various other factors, including personal experiences and social influences may also impact the decision to seek or to avoid care, which were not captured in our questionnaire. Additionally, the effect of Thuisarts.nl on healthcare utilization among individuals with chest pain should be studied, focusing on measurable outcomes, such as reductions in GP consultations or ED visits.

## Conclusions

Chest pain is a common reason for patients to seek online health information. Thuisarts.nl used for chest pain is considered helpful by most visitors, primarily a low-risk group. The website should be tailored to this group, while still addressing acute situations. It appears that people whose pain seems to be of cardiac origin are more likely to contact a healthcare provider immediately, whereas those with other non-life-threatening causes of chest pain tend to wait and monitor their symptoms longer. However, Thuisarts.nl is not easily understood by everyone and could be improved for people with low literacy, incorporating illustrations and videos. These findings highlight an opportunity to enhance early-stage healthcare support, ultimately contributing to a more efficient and sustainable healthcare system for chest pain patients, potentially reducing GP consultations and ED visits.

## Supplementary Information

Supplementary Material 1.

## Data Availability

The datasets generated and/or analysed during the current study are not publicly available because they were collected within a closed environment (via Thuisarts) and contain personal data, but are available from the corresponding author on reasonable request.

## Abbreviations

CAD: Coronary artery disease
CI: Confidence interval
ED: Emergency department
EDC: Electronic Data Capture
GP: General practitioner
HBM: Health Belief Model
NHG: Dutch College of General Practitioners
