# Supplementary material for: Navigating chest pain online: who uses health information and what actions follow?
Source: BMC Prim Care. 2025 Nov 25;27:1. doi: 10.1186/s12875-025-03110-7 (PMC12764119; doi:10.1186/s12875-025-03110-7)
Supplement: Supplementary file 1 — Supplementary Material 1. [file 12875_2025_3110_MOESM1_ESM.docx]

**Supplementary File: Questionnaire Thuisarts.nl (English Version)**

**Questions Thuisarts.nl – call-to-action button with a link to the questionnaire for every visitor on the pages “I have chest pain” and “I have angina”**

You have been looking for information about chest pain or heart-related symptoms. We want to help you—and others—find reliable information. We are conducting this research from the Leiden University Medical Center (LUMC). Would you be willing to answer a few questions to help us?

1. What is your age? Click or tap here to enter text.

If you are under 18 years of age, you do not need to complete the questionnaire.
If you are 18 or older, question 2 will appear next.

2. Did you visit this website for yourself or for someone else?

For myself

For someone else

If you selected "for someone else", you do not need to complete the rest of the questionnaire. If you selected "for myself", question 3 will appear next.

3. You wanted to know what to do when you had chest pain. Did the information on the Thuisarts.nl website help you?

3a Yes, I immediately called my GP or the out-of-hours clinic to make an appointment.

3b Yes, I will contact my own GP within a few days.

3c Yes, I am waiting to see how things go.

3d The information helped me to some extent.

3e No, the information did not help me.

3a – proceed to question 4

3b – proceed to question 5

3c – proceed to question 6

3d – proceed to question 7

3e – proceed to question 8

**Question 4**

4.1 What information made you decide to call your GP or the out-of-hours clinic immediately? *(You can select more than one answer)*

I read the text “Call your GP or the out-of-hours clinic directly in the following cases:”. This made me decide to call my GP or the out-of-hours clinic immediately.

I read the text “Call 112 immediately if...”. This made me decide to call my GP or the out-of-hours clinic immediately.

I read the text “What do you experience with anginal chest pain?”. This made me decide to call my GP or the out-of-hours clinic immediately.

My chest pain seemed to match the description of pain related to:

*(You can select more than one answer)*

Musculoskeletal pain

Stomach and oesophagus

Stress, anxiety, or depression

Heart

Lungs

Skin

I don’t know

4.2a Why did you call your GP or the out-of-hours clinic immediately? *(You can select more than one answer)*

The information made me take my symptoms more seriously.

The information helped me understand the potential risks to my health.

The information made me more concerned about the cause of my symptoms.

The information confirmed that my suspicions or concerns were correct.

Other, namely: Click or tap here to enter text.

If you selected 4.2a: “The information made me more concerned about the cause of my symptoms,” please also answer question 4.2b.

4.2b How concerned were you about your chest pain after reading the information? Please rate it from 0 to 10.

|  | 0 | 1 | 2 | 3 | 4 | 5 | 6 | 7 | 8 | 9 | 10 |  |
| --- | --- | --- | --- | --- | --- | --- | --- | --- | --- | --- | --- | --- |
| No concern |  |  |  |  |  |  |  |  |  |  |  | I have never been this concerned. |

**Question 5**

5.1 What information made you decide to contact your own GP within a few days? *(You can select more than one answer)*

I read the text “Call your GP or the out-of-hours clinic directly in the following cases:”. This made me decide to contact my GP within a few days.

I read the text “What do you experience with anginal chest pain?”. This made me decide to contact my GP within a few days.

My chest pain seemed to match the description of pain related to:

*(You can select more than one answer)*

Musculoskeletal

Stomach and oesophagus

Stress, anxiety, or depression

Heart

Lungs

Skin

I don’t know

5.2 Why are you contacting your own GP within a few days?

5a I didn't want to call at first, but the information on Thuisarts.nl has made me decide to contact my GP within a few days.

5b I wanted to call my GP or the out-of-hours clinic immediately, but the information on Thuisarts.nl has made me wait and contact my GP within a few days instead.

5c The information confirmed that my suspicions or concerns were correct.

If you selected 5a, please answer question 5.3.

If you selected 5b, please answer question 5.4.

5.3a Why did you decide to call your GP after all? *(You can select more than one answer)*

The information made me take my symptoms more seriously.

The information helped me understand the potential risks to my health.

The information made me more concerned about the cause of my symptoms.

Other, namely: Click or tap here to enter text.

If you selected 5.3a: “The information made me more concerned about the cause of my symptoms,” please also answer question 5.3b.

5.3b How concerned were you about your chest pain after reading the information? Please rate it from 0 to 10.

|  | 0 | 1 | 2 | 3 | 4 | 5 | 6 | 7 | 8 | 9 | 10 |  |
| --- | --- | --- | --- | --- | --- | --- | --- | --- | --- | --- | --- | --- |
| No concern |  |  |  |  |  |  |  |  |  |  |  | I have never been this concerned. |

5.4a Why did you decide not to call your GP or the out-of-hours clinic immediately? *(You can select more than one answer)*

The information helped me understand the potential risks to my health.

The information made me less concerned about the cause of my symptoms.

Other, namely: Click or tap here to enter text.

If you selected 5.4a: “The information made me more concerned about the cause of my symptoms,” please also answer question 5.4b.

5.4b How concerned were you about your chest pain after reading the information? Please rate it from 0 to 10.

|  | 0 | 1 | 2 | 3 | 4 | 5 | 6 | 7 | 8 | 9 | 10 |  |
| --- | --- | --- | --- | --- | --- | --- | --- | --- | --- | --- | --- | --- |
| No concern |  |  |  |  |  |  |  |  |  |  |  | I have never been this concerned. |

**Question 6**

6.1 What information made you decide not to contact a doctor yet? *(You can select more than one answer)*

I read the text “Call your GP or the out-of-hours clinic directly in the following cases.” This made me decide that I did not need to call a doctor immediately.

I read the text “Call 112 immediately if...”. This made me decide that I did not need to call a doctor immediately.

I read the text “What do you experience with anginal chest pain?”. This made me decide that I did not need to call a doctor.

My chest pain seemed to match the description of pain related to:

*(You can select more than one answer)*

Musculoskeletal

Stomach and oesophagus

Stress, anxiety, or depression

Heart

Lungs

Skin

I don’t know

6.2a Why did you decide not to call? *(You can select more than one answer)*

The information made me take my symptoms less seriously.

The information helped me understand the potential risks to my health.

The information made me less concerned about the cause of my symptoms.

The information made me realise that my suspicions or concerns were incorrect.

Other, namely: Click or tap here to enter text.

If you selected 6.2a: “The information made me more concerned about the cause of my symptoms,” please also answer question 6.2b.

6.2b How concerned were you about your chest pain after reading the information? Please rate it from 0 to 10.

|  | 0 | 1 | 2 | 3 | 4 | 5 | 6 | 7 | 8 | 9 | 10 |  |
| --- | --- | --- | --- | --- | --- | --- | --- | --- | --- | --- | --- | --- |
| No concern |  |  |  |  |  |  |  |  |  |  |  | I have never been this concerned. |

**Question 7**

7.1 How did the information help you?

Click or tap here to enter text.

7.2 What information helped you make this decision? *(You can select more than one answer)*

I read the text “Call your GP or the out-of-hours clinic directly in the following cases.”

I read the text “Call 112 immediately if...”

I read the text “What do you experience with anginal chest pain?”.

My chest pain seemed to match the description of pain related to:

*(You can select more than one answer)*

Musculoskeletal

Stomach and oesophagus

Stress, anxiety, or depression

Heart

Lungs

Skin

I don’t know

7.3 How concerned were you about your chest pain after reading the information? Please rate it from 0 to 10.

|  | 0 | 1 | 2 | 3 | 4 | 5 | 6 | 7 | 8 | 9 | 10 |  |
| --- | --- | --- | --- | --- | --- | --- | --- | --- | --- | --- | --- | --- |
| No concern |  |  |  |  |  |  |  |  |  |  |  | I have never been this concerned. |

**Question 8**

8.1 Why didn’t the information help you decide what to do about your chest pain? *(You can select more than one answer)*

I didn’t understand the information well.

The pain I felt didn’t fully match any of the descriptions of pain on Thuisarts.nl.

I wasn’t sure if my pain was serious enough.

I wasn’t sure if my pain had lasted long enough.

The reasons to call my GP or 112 didn’t apply to me, but I also didn’t know what else I should do.

Other, namely: Click or tap here to enter text.

8.2 How concerned were you about your chest pain after reading the information? Please rate it from 0 to 10.

|  | 0 | 1 | 2 | 3 | 4 | 5 | 6 | 7 | 8 | 9 | 10 |  |
| --- | --- | --- | --- | --- | --- | --- | --- | --- | --- | --- | --- | --- |
| No concern |  |  |  |  |  |  |  |  |  |  |  | I have never been this concerned. |

1. How would you describe the pain?

☐ Stabbing or sharp

☐ Tight, squeezing, or pressure-like

☐ Pain when breathing

☐ I can’t describe the type of pain clearly.

☐ Other

1. Did the pain start due to something, or did the pain worsen due to something? *(You can select more than one answer)*

No, the pain started suddenly without doing anything and stayed the same throughout.

Yes, the pain started or worsened with physical exertion (e.g., exercising, walking, cleaning, gardening).

Yes, the pain started or worsened with deep breathing.

Yes, but the pain worsened due to something other than exertion or deep breathing.

1. Did the pain lessen when you sat down and rested?

☐ No, the pain stayed the same.

☐ No, the pain even got worse.

☐ Yes, the pain got less.

☐ Yes, the pain completely went away.

1. Can you describe how severe the pain was? Compare it to how you normally feel.

☐ I could do everything as I usually do.

☐ I had little pain, I could almost do everything.

☐ The pain made it harder to do things.

☐ I was bothered by the pain with everything I did.

☐ The pain was so severe that I couldn’t do anything.

1. How did the symptoms start?

Slowly

Quickly or suddenly

1. Did you have any other symptoms besides chest pain? *(You can select more than one answer)*

No, I only had chest pain.

Yes, I was also sweating.

Yes, I was also feeling nauseous.

Yes, I was also short of breath and breathing quickly.

Yes, but I had other symptoms besides sweating, nausea, or shortness of breath.

1. Did you have a fever in the days before visiting Thuisarts.nl?

☐ Yes

☐ No

1. What is your gender?

Male

Female

Other or I’d prefer not to say

1. Have you had any heart or blood vessel diseases? *(You can select more than one answer)*

No, I haven’t.

Yes, a heart attack (myocardial infarction)

☐ Yes, angina (chest pain from narrowed heart arteries)

☐ Yes, a stroke (TIA or CVA)

☐ Yes, peripheral artery disease (narrowed arteries in the legs)

☐ Yes, diabetes

☐ Yes, high blood pressure (hypertension)

☐ Yes, but different from the diseases listed above.

I don’t know.

1. Do you take medication every day for a heart or blood vessel disease?

Yes

No

I don’t know
